# A case of septic arthritis caused by *Capnocytophaga canimorsus* in an HIV patient

**DOI:** 10.1099/acmi.0.000368

**Published:** 2022-06-15

**Authors:** Domingo Fernández Vecilla, Cristina Aspichueta Vivanco, Itziar Angulo López, Josu Mirena Baraia-Etxaburu Artetxe, Francesco Renzi, José Luis Díaz de Tuesta del Arco

**Affiliations:** ^1^​ Basurto University Hospital, Avenida Montevideo n°18, 48013, Bilbao (Vizcaya), Spain; ^2^​ Namur Research Institute for Life Sciences, Research Unit in Biology of Microorganisms, University of Namur, 61 Rue de Bruxelles, 5000 Namur, Belgium

**Keywords:** zoonotic infections, *Capnocytophaga canimorsus*, septic arthritis, native joint, HIV, virulence factors

## Abstract

Invasive infections caused by *Capnocytophaga canimorsus *, a Gram-negative rod found in the oral cavity of healthy dogs and cats, are rare but they are increasing worldwide. We report a case of septic arthritis in a native knee joint due to this micro-organism. A 57-year-old man, with a well-controlled chronic HIV infection, attended the Emergency Department because of left knee pain and shivering without measured fever. A knee arthrocentesis and a computed tomography scan were performed, revealing septic arthritis with collections in the left leg posterior musculature. He was admitted to the Infectious Diseases Department for antibiotic treatment. Initial synovial fluid was inoculated in blood culture bottles, and the anaerobic one was positive after 63 h. Gram stain revealed fusiform Gram-negative rods, identified as *C. canimorsus* by matrix-assisted laser desorption/ionization time-of-flight mass spectrometry (MALDI-TOF) directly from the bottle. Identification was confirmed by 16S rRNA sequencing and serotyping was performed by PCR, with serovar A as the outcome. Due to an unfavourable clinical course, the patient required two surgical cleanings and after appropriate antibiotic treatment he was discharged 2 months later.

## Introduction

*Capnocytophaga canimorsus* is a slow-growing, facultative anaerobic, Gram-negative rod that belongs to the family *Flavobacteriaceae *. It is found as commensal oropharyngeal flora in healthy dogs and cats. It can be transmitted to humans by exposure to these animals, usually by bites, licks, scratches or even close contact. *C. canimorsus* is well documented as causing severe infections with a mortality of ~30 % [1]. Typically, these infections are characterized by sepsis, which can be complicated by septic shock or disseminated intravascular coagulation (DIC); meningitis, endocarditis and less frequently osteoarticular infections have also been described [2, 3]. We report a case of *C. canimorsus-*related septic arthritis in a native knee.

## Case

A 57-year-old man presented to the Emergency Room with pain in the left knee, difficulty walking and fever for 3 days. He had been an intravenous drug user, with chronic hepatitis C and HIV infections diagnosed 30 years ago. He had good immune control (undetectable viral load and CD4=879 mm^−3^). Among other background information, he wore bilateral hip prosthesis and suffered from mixed sensorimotor polyneuropathy (ethanol–HIV). The patient lived with a dog but he did not refer any bites or scratches, only licks.

**Fig. 1.**
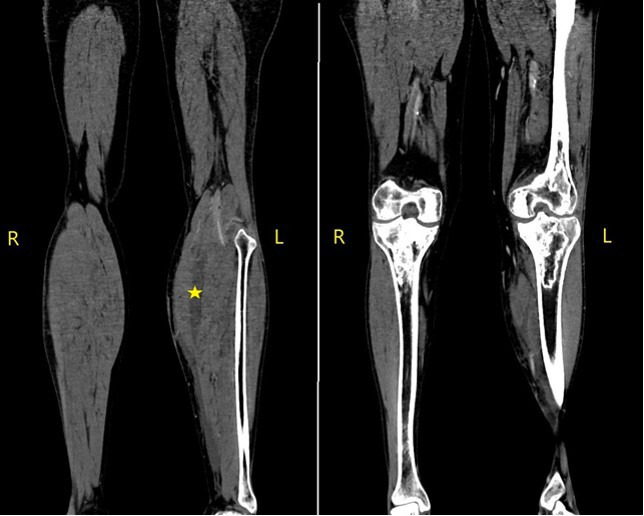
Lower extremity CT scan with intravenous contrast. Signs of inflammatory arthritis with collections that extend primarily between the medial calf and soleus in the left leg. Deep venous thrombosis is not demonstrated. There is no evidence of osteomyelitis despite the findings of bone infarction in both knees.

Initial examination revealed intense pain, erythema and swelling in the left knee and thigh, without external cellulitis signs. An arthrocentesis was performed, showing an inflammatory liquid with 44 300 leucocytes mm^−3^ (80 % neutrophils). Blood test values were 12 700 leucocytes mm^−3^, with 80 % of neutrophils and a reactive C protein value of 262 mg l^−1^. The patient was admitted to the Infectious Disease Service and treated empirically with intravenous ceftriaxone and cloxacillin (2 g/50 ml every 24 h and 1 g/50 ml every 6 h, respectively). In spite of treatment, the patient continued with severe pain and a computed tomography (CT) scan of the lower extremities was performed (Fig. 1). It showed collections between the gemellus and soleus muscles, as well as septic arthritis signs.

## Methodology

The initial synovial fluid obtained in the Emergency Department was cultured in BD chocolate agar plates, BD trypticase soy agar (TSA) with 5 % sheep blood plates for aerobic incubation and BD *Brucella* agar plates with 5 % sheep blood for anaerobic incubation (Becton Dickinson, Franklin Lakes, NJ, USA). It was also inoculated into one set of blood culture bottles (BACTEC, Becton Dickinson, Franklin Lakes, NJ, USA). Bottle direct identification was performed by matrix-assisted laser desorption/ionization time-of-flight mass spectrometry (MALDI-TOF MS) (Bruker Daltonics) after the following in-house procedure had been completed: transfer 1 ml from a positive bottle, centrifuge for 2 min at 13 000 r.p.m., discard the supernatant, apply the pellet to the polished steel target plate and, once dried, overlay with 1 μl of matrix solution (alfa-cyano-4-hydroxycinnamic acid). Antibiotic susceptibility testing was performed using Liofilchem MIC Test Strips on Mueller–Hinton agar plates with 5 % sheep blood (Becton Dickinson, Franklin Lakes, NJ, USA) incubated in 5–10 % CO_2_ atmosphere.

## Results

**Fig. 2.**
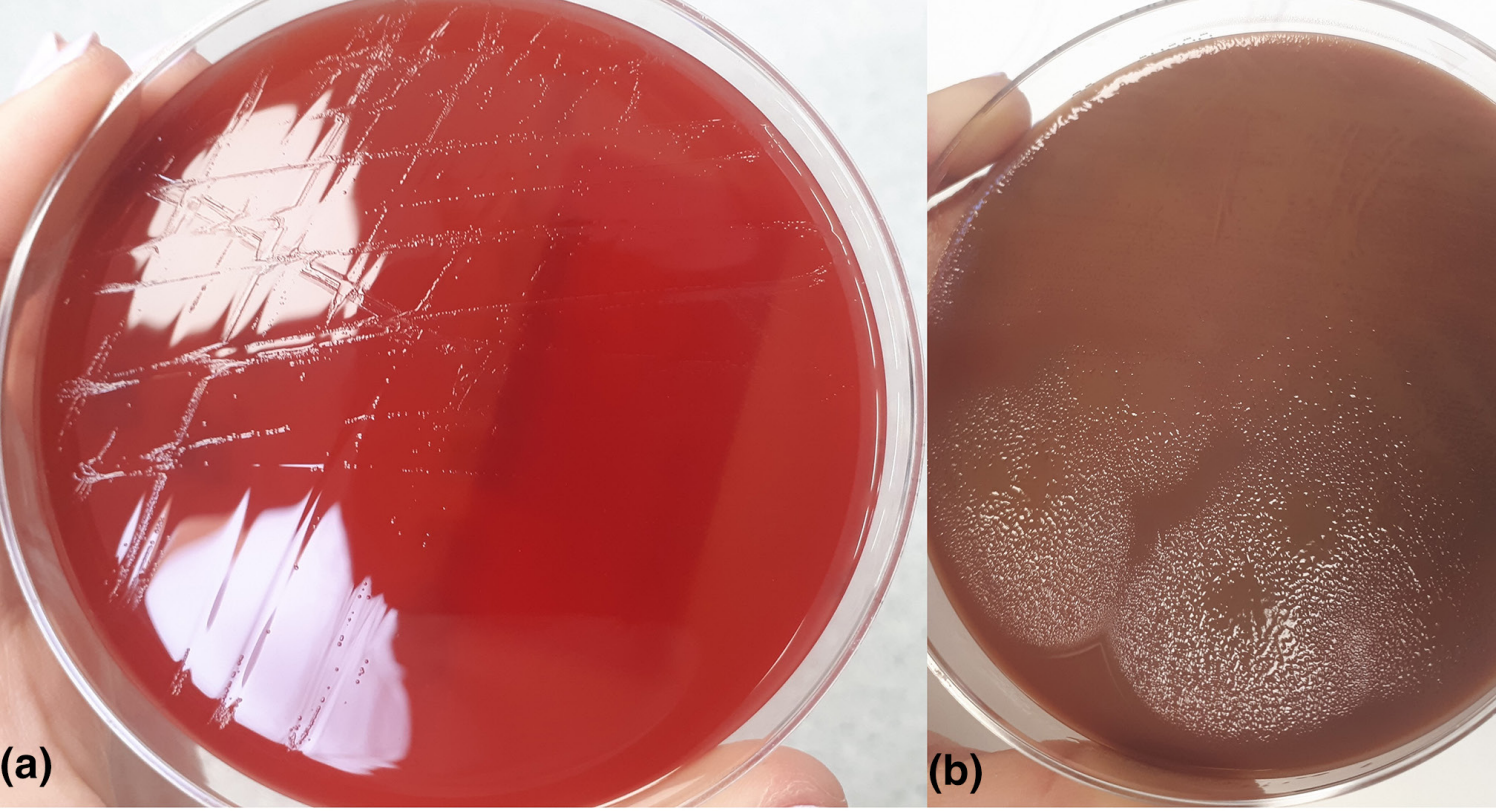
(a) *Capnocitophaga canimorsus* with small, transparent colonies after 96–120 h of incubation in TSA agar with 5 % sheep blood (Becton Dickinson, Franklin Lakes, NJ, USA) that acquire a greyish coloration some days after. (b) colonies of *Capnocytophaga sputigena* with a sliding movement in chocolate agar (Becton Dickinson, Franklin Lakes, NJ, USA). This image exemplifies the gliding mobility of *Capnocytophaga* spp. in culture.

After 5 days, no growth was observed on agar plates from the direct cultures of synovial fluid. The anaerobic bottle was positive after 63 h of incubation and the aerobic one after 4 days. Gram stain revealed curved Gram-negative bacilli that were subcultured on chocolate and *Brucella* agar plates. Bottle direct bacterial identification was performed by MALDI-TOF MS and *C. canimorsus* was identified with a score of 1.89. After 8 days, there was a veil-like growth in the TSA with 5 % sheep blood agar (Fig. 2a). A few days later, greyish colonies were observed, and were identified by MALDI-TOF as *C. canimorsus* with a score of 2.34 (Supplementary Material, available in the online version of this article). The strain was submitted to a reference centre, the Research Unit in Biology of Microorganisms at the University of Namur (Belgium), which confirmed *C. canimorsus* identification by 16S rRNA sequencing, following Mally *et al.*’s protocol [4]. Capsular typing was also performed by PCR, according to Hess *et al.* (Supplementary Material), showing the isolate to be a serovar A [5–7]. The antibiotic susceptibility results are shown in Table 1. Antibiotic susceptibility was determined according to the European Committee on Antimicrobial Susceptibility Testing (EUCAST) 2020 PK/PD as well as the antimicrobial MIC breakpoints of the HACEK micro-organism group established by the Clinical and Laboratory Standards Institute (CLSI) (in the ‘infrequently isolated or fastidious bacteria’ guidelines) [8, 9].

**Table 1.** Antimicrobial susceptibility results

Antibiotic	E-test/MICs (mg dl^−1^)	Disk diffusion/inhibition zone diameters (mm)
Amoxicillin+clavulanic	<0.016	28
Amoxicillin	0.016	26
Ceftriaxone	<0.064	27
Meropenem	0.003	35
Clindamycin	<0.016	25
Tetracycline	0.125	23
Piperacillin+tazobactam	<0.016	30
Trimethoprim/sulfamethoxazole	0.064	25

## Outcome

On the fifth day of admission, the patient was submitted to surgical cleaning and drainage of the collection by arthrotomy. When the microbiologist identified and reported *C. canimorsus* in initial joint fluid, the antibiotic treatment was changed from centriaxone and cloxacillin to piperacillin/tazobactam (4 g/0.5 g every 8 h IV). Due to an unfavourable clinical course with worsening pain and persistent low-grade fever for 4 weeks, the patient required a second cleaning 13 days later, and a large haematoma was observed, which was drained. One month later, antibiotic therapy was modified to ceftriaxone (2 g every 24 h IV) for 10 days. Finally, the treatment was de-escalated to orally administered clindamycin (600 mg every 8 h). Two months after admission, he was discharged with a good clinical outcome, completing 9 weeks of antibiotic treatment.

## Discussion

Very few cases of osteoarticular infections caused by *Capnocytophaga canimorsus* have been described in the literature. It has been reported in prosthetic hip and knee joint infections in immunocompetent patients as well as acute tenosynovitis of the ankle in an immunosuppressed patient [10–14].

*C. canimorsus* is a common host of the dog’s oral flora, but the number of human infections remains quite low, suggesting that other factors are necessary to produce infection. As the main virulence factors, this micro-organism has a lipooligosaccharide, a lipid A and a capsular polysaccharide (CPS), composed of the same O-antigen repeating units. Currently, nine serovars have been described by Renzi *et al.* (A, B, C, D, E, F, G, H, I and non-capsulated) in human isolates. The A, B and C serovars, despite being infrequent in dogs (<8%), appear to be more virulent and represent the great majority of human isolates, like our strain, which belongs to serovar A [15]. CPS could play a major role in *C. canimorsus* pathogenesis, especially at the onset of the infection, when the innate immune system tries to mitigate it. CPS would confer protection against phagocytosis, the bactericidal effect of serum and antimicrobial peptides, which are part of the innate immune response. This is probably the reason why these bacteria could lead to severe complications, such as disseminated intravascular coagulation (DIC), meningitis or fulminant sepsis, even in immunocompetent patients or those without medical backgrounds [16–19].

The main risk factors for increased susceptibility to *C. canimorsus* infections are male sex, age over 50 years, splenectomy, chronic alcohol consumption, tobacco smoking, diabetes mellitus and use of immunosuppressive therapies [2]. However, more than 40 % of the patients have no evident risk factor [16].

*C. canimorsus* infections may have been underestimated because of the difficulty of isolating the bacteria due to the slow and unique growth media requirements. Improved culture media, especially inoculation of the sample into blood culture bottles, probably contributes to increased detection [2]. This fact was crucial in our case, where direct seeding of the synovial fluid produced a negative result, while inoculation into blood culture bottles allowed us to determine the aetiological diagnosis in <3 days. It has also been suggested that the the increase in *C. canimorsus* cases is mainly due to improved techniques for bacterial identification, such as the generalization of MALDI-TOF MS [20, 21]. Our diagnosis was based on the blood culture bottle direct MALDI-TOF MS method as we previously described. Therefore, we believe it represents an accurate, rapid and inexpensive tool for detection of this fastidious pathogen. 16S rRNA gene sequencing or complete genome sequencing performed directly on a clinical sample are other useful techniques to decrease time to definitive diagnosis [22, 23].

*C. canimorsus* is broadly susceptible to all beta-lactams, clindamycin and tetracycline [1, 24–26]. Some strains could occasionally produce beta-lactamase enzymes, although it is very infrequent in this species [2]. The nitrocefin test can be used to detect beta-lactamases-producing strains. Most isolates are resistant or variably sensitive to aminoglycosides, trimethoprim/sulfamethoxazole and polymyxins [1, 27, 28]. Treatment options include penicillin/beta-lactamase combinations, third-generation cephalosporins, carbapenems, clindamycin and doxycycline. The results from the susceptibility tests on our isolate match those from previous reports [24, 29, 30]. In a previous case report of a periprosthetic joint infection produced by *C. canimorsus *, the indicated antibiotic treatment was ceftriaxone for 6 weeks [11]. In our case, in addition to antibiotic treatment two surgical cleanings were required due to an unfavourable course.

In conclusion, the incidence of *C. canimorsus* infection is expected to increase over time because of reliable new diagnostic methods, a more susceptible population and the increasing popularity of pet ownership. This case reinforces the importance of clinicians being aware that *C. canimorsus* could be the causative agent of a variety of invasive infections in patients with close dog contact.

## Supplementary Data

Supplementary material 1
