# Supplementary material for: A case of septic arthritis caused by Capnocytophaga canimorsus in an HIV patient
Source: Access Microbiol. 2022 Jun 15;4(6):acmi000368. doi: 10.1099/acmi.0.000368 (PMC9394666; doi:10.1099/acmi.0.000368)
Supplement: Supplementary material 1 [file acmi-4-368-s001.pdf]

Supplementary material

1. MALDI-TOF mass spectrometry results:

The identification score from the studied sample (synovial fluid) is 2.35 with *Capnocytophaga canimorsus* as result. A score ≥ 2.00 is reliable for species level identification, whereas a score ≥ 1.70 is reliable to genus level identification. The MALDI-TOF mass spectometer may identify different species from the *Capnocytophaga* genus: *Capnocytophaga canimorsus*, *Capnocytophaga cynodegmi* , *Capnocytophaga gingivalis*, *Capnocytophaga granulosa*, *Capnocytophaga haemolytica*, *Capnocytophaga ochracea* and *Capnocytophaga sputigena*.

Bruker Daltonik MALDI Biotyper
Resultados de la identificación

BRUKER

Información sobre el proyecto:

Identificador de la secuencia:	220208-1056-1011014058
Comentario:	
Operador:	tof-user@MBT-WIN10
Fecha/hora de creación de la secuencia:	2022-02-08T10:57:19.714
Número de pruebas:	3
Tipo:	Estándar
Validación:	inexistente
Posición de validación:	
ID de instrumento:	8604832.05129
Versión del servidor:	4.1.100 (PYTH) 174 2019-06-158_01-16-09

Resumen de resultados

Analyte Nombre	Analyte ID	Organismo (mejor candidato)	Puntuación Valor	Organismo (segundo mejor candidato)	Puntuación Valor
D6 (+++) (A)	cani (Estándar)	Capnocytophaga canimorsus	2.08	Capnocytophaga canimorsus	2.08
D7 (+++) (A)	cani (Estándar)	Capnocytophaga canimorsus	2.35	Capnocytophaga canimorsus	2.33
D8 (-) (A)	cani (Estándar)	**no se han encontrado picos**	0.00	**no se han encontrado picos**	0.00

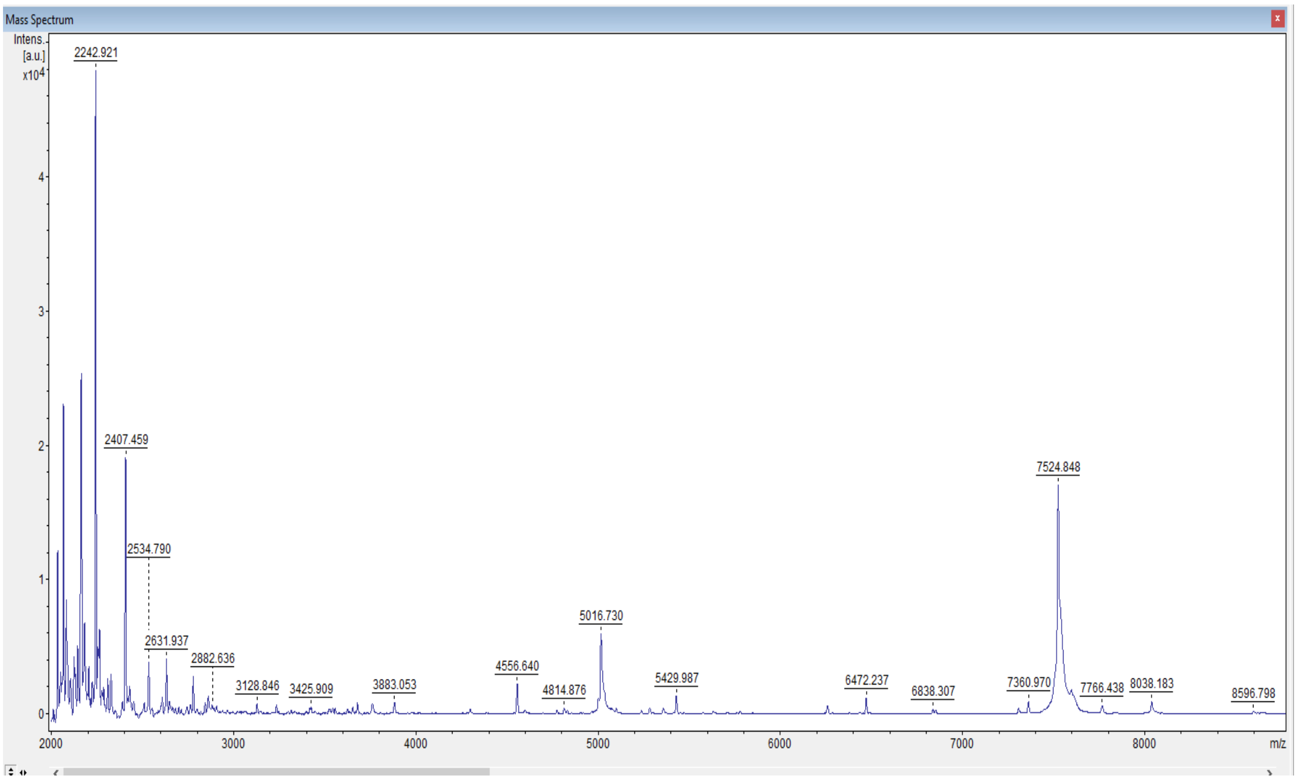

2. <u>16S rRNA sequencing:</u>

Protocol: a single colony was resuspended in 100 µl of double distilled water and heated for 15 min at 98 °C. Five µl were used as template for amplification of 1.07 kb of the 16S rRNA gene. Primers 27F (5'-agagtttgatcctggctcag-3') and 1100R (5'-gggttgcgctcgttg-3'), binding in the conserved region, were used at 0.4 µM concentration with 200 µM dNTP and 1 U Taq polymerase (NEB). PCR was carried out for 5 initial cycles (90º C for 30 s, 60º C for 2 min, 72º C for 3 min) in which the annealing temperature was reduced by 1.5º C/cycle, followed by 30 cycles (94º C for 30 s, 52º C for 90 s, 72º C for 3 min) and final elongation for 10 min at 72º C.

The 1.1 kb PCR product was extracted from a 1% agarose gel by NucleoSpinÒ (Machery Nagel). The PCR product was sequenced with primers 27F, 685R (5'-tctacgcatttcaccgctac-3') and 1100R following the protocol proposed by Mally M et al [1].

Sequences were aligned with Clustal Omega and consensus sequence submitted to Blastn suite.

The sequence obtained was the following:

CTAACACATGCAAGTCGAGGGGTAGGGTGCTTCGGCACTTGAGACCGGCGCACGGGTG
CGTAACACGTGTACAATCTACCTTTTGCTAAGGGATAGCCCGAAGAAATTTGGATTAAT
ACCTTATAGTATTGTTTGGTGGCATCACTGAATAATTAAAGCTCTGGTGGCAAAAGATG
AGTACGCGTCCCATTAGCTAGTTGGTGTGGTAACGGCATACCAAGGCTACGATGGGTA
GGGGTCCTGAGAGGGAGGTCCCCCACACTGGTACTGAGACACGGACCAGACTCCTACG
GGAGGCAGCAGTGAGGAATATTGGACAATGGTCGGAAGACTGATCCAGCCATGCCGC
GTGCAGGATGACGGCCTTATGGGTTGTAAACTGCTTTTATACAGGAAGAATAAGGTCT

ACGAGTAGATTGATGACGGTACTGTATGAATAAGCATCGGCTAACTCCGTGCCAGCAG
CCGCGGTAATACGGAGGATGCGAGCGTTATCCGGAATCATTGGGTTTAAAGGGTCCGT
AGGCGGGCTTATAAGTCAGAGGTGAAAGCACTGAGCTCAACTGAGTAACTGCCTTTGA
AACTGTAGGTCTTGAATGTTTGTGAAGTAGCTGGAATGTGTAGTGTAGCGGTGAAATG
CATAGATATTACACAGAAC

The 16S sequence was not submitted to Gene Bank. This is the 16S consensus sequence we obtained and submitted to Blastn. We did not do phylogenesis.

3. <u>Capsular typing:</u>

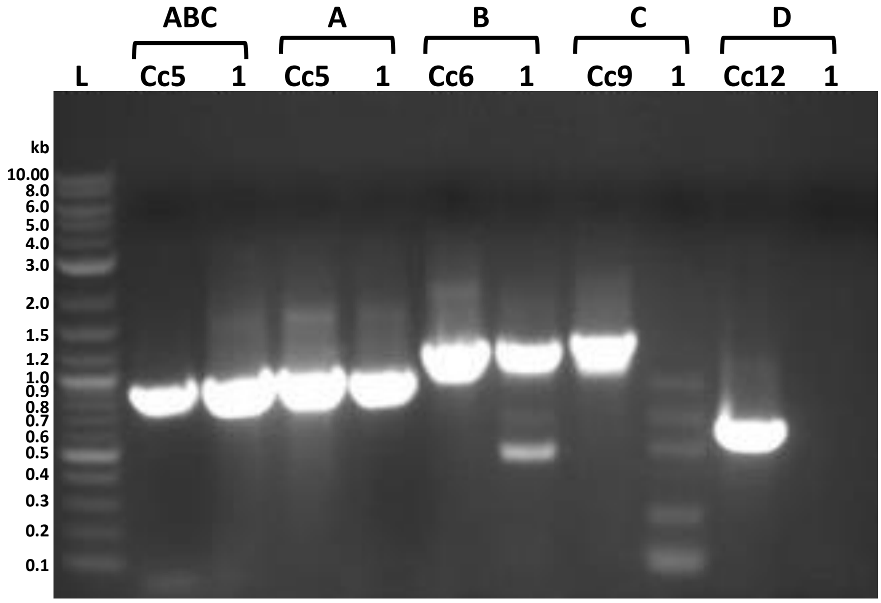

Protocol: a single colony was resuspended in 100 µl of double-distilled water and heated for 15 min at 98°C. Two microliters were used as the template for amplification. PCR detection was performed using the Promega Go Taq G2 polymerase (Madison, WI, USA) under the following conditions: initial denaturation at 95°C for 4 min, followed by 35 cycles of denaturation at 95°C for 30 s, annealing at 52°C for 45 s, extension at 72°C for 1 min and 30 s, and a final extension at 72°C for 7 min as proposed by Hess *et al.* [2].

Primers used:
SeroA-fw CATACCATGGGAAAAAAAGTACCAATAGTTTTTATATTTAACC
SeroA-rev CCGCTCGAGTCATTTTTTTATCTTTTTTAATATATTCCAC

SeroB-fw CATACCATGGGAATTAACAAAATTCTAATAG
SeroB-rev CCGCTCGAGTTATTTTTTATTTTCATTAG
SeroC-fw GGCGTATATCGTTGCTATTTTGTATG
SeroC-rev CTATTAATATTTTCATTGTACACCACTTC
SeroD-fw GATTTAAAAAATATAGTATTTTAGGAATTATCG
SeroD-rev CTATACTTGTTCCCACTTTTTAGTTTC
SeroABC-fw CTTGGTTAGGTAAAGTTGCCTTAC
SeroABC-rev CAACATTTCTCCCATCTTATAATCCC

Capsular typing by PCR of the *C. canimorsus* strain we isolated in this study. Capsular serovars A to D detection by PCR was performed as described by Hess *et al.* L: 2-Log DNA ladder; 1: *C. canimorsus* strain isolated in this study; Cc5: *C. canimorsus* strain 5 serovar A; Cc6*: C. canimorsus* strain 6 serovar B; Cc9: *C. canimorsus* strain 9 serovar C; Cc12: *C. canimorsus* strain 12 serovar D. The *C. canimorsus* strain we isolated in this study is positive for PCR ABC, A and B and thus belongs to capsular serovar A (PCR B detects strains belonging to serovar B and some belonging to A).

4. References:

1. Mally M, Cornelis GR. Genetic tools for studying *Capnocytophaga canimorsus*. Appl Environ Microbiol. 2008 Oct;74(20):6369-77. doi: 10.1128/AEM.01218-08.

2. Hess E, Renzi F, Koudad D, Dol M, Cornelis GR. Identification of Virulent Capnocytophaga canimorsus Isolates by Capsular Typing. J Clin Microbiol. 2017 Jun;55(6):1902-1914. doi: 10.1128/JCM.00249-17.
